# The role of hyaluronan modification in the etiopathogenesis of gastric cancer

**DOI:** 10.1590/1806-9282.20241087

**Published:** 2024-12-16

**Authors:** Ahmet Turan Durak, Hızır Yakup Akyıldız, Pınar Altın Çelik, Metin Aytekin

**Affiliations:** 1 T.R. Ministry of Health Abant İzzet Baysal State Hospital, Department of General Surgery – Bursa, Turkey.; 2 Erciyes University, Department of General Surgery – Kayseri, Turkey.; 3 Erciyes University, Department of Medical Biology – Kayseri, Turkey.; 4 Cleveland Clinic, Department of Pathobiology – Cleveland (OH), United States.

**Keywords:** Hyaluronan, Gastric cancer, Heavy chain, Extracellular matrix

## Abstract

**OBJECTIVE:**

Gastric cancer is the second most common cancer in the world, accounting for 650,000 deaths per year, and is observed in approximately 10% of the patients diagnosed. Extracellular matrix abnormalities have been documented in gastric cancer patients. The aim of our study was to understand the role of high levels of hyaluronan, an extracellular matrix glycosaminoglycan, and its mechanistic role in gastric cancer pathobiology.

**METHODS:**

Blood samples were collected from 82 gastric cancer patients and 41 healthy volunteers. Hyaluronan measurements were performed with the help of commercially purchased enzyme-linked immunosorbent assay kits. Gastric cancer (n=27) and healthy (n=29) tissue specimens were obtained after surgery and aliquoted for Western blot, immunofluorescence, and messenger RNA expression analysis.

**RESULTS:**

Increased hyaluronan levels were detected in the blood of cancer patients compared to controls [plasma hyaluronan levels mg/dL (mean±SD): gastric cancer (n=82) 549.80±155.68, and healthy control (n=41) 27.21±4.95 (p<0.044)]. In addition, intense hyaluronan binding protein staining was observed in gastric cancer tissues, while tumor necrosis factor-inducible gene 6 messenger RNA expression was found to be significantly increased in gastric cancer tissues compared to healthy controls [tumor necrosis factor-inducible gene 6 messenger RNA expression: gastric cancer (n=27) 7.09±1.94 and healthy control (n=29) 3.20±0.67 (p=0.048)] according to the immunofluorescence staining.

**CONCLUSION:**

The high hyaluronan levels in gastric cancer patients and the detection of increased messenger RNA levels of the tumor necrosis factor-inducible gene 6 enzyme in gastric cancer tissue, supporting a possible hyaluronan modification, suggest that this abnormality may have an important role in the formation of gastric cancer.

## INTRODUCTION

Gastric cancer is the second most common type of cancer in the world^1^. The incidence of gastric cancer is known to increase after the age of 50 years and is reported to be equally prevalent for both sexes at the age of 70 years^2^.

Extracellular matrix (ECM) has important roles in cell proliferation and migration. There are studies showing that some cancer types, including gastric cancer, are associated with ECM abnormalities^3^. Identifying matrix abnormalities in gastric cancer will help us understand the course of the disease. Hyaluronan (HA), which is a free glycosaminoglycan chain, is also found in the stomach. In addition, it has been reported that the degraded products of HA also stimulate angiogenesis^4^.

The inter-alpha-inhibitor (IαI) complex, which is a serum protein, consists of a proteoglycan called "bikunin" and two "heavy chain" molecules covalently linked by a chondroitin sulfate chain. It is secreted into the blood in high concentrations by the liver and was first found in urine and serum as a trypsin inhibitor^5^. The functions of heavy chains connecting to IαI are still not elucidated. It is thought that only three forms of the heavy chain molecule, which has six different types, may be associated with IαI and HA^6^. Under the cofactor of the tumor necrosis factor-inducible gene 6 (TSG-6) enzyme, these heavy chain molecules form a skeleton by binding to HA, and this structure is known to be involved in the calling of inflammatory cells^7^. We believe that HA may be involved in carcinogenesis. If this role is proven, then an alternative method can be developed to prevent the occurrence of the disease by blocking HA production.

Comparison of HA levels in cancerous and healthy tissues has been studied before at the level of different organs, and high levels of HA in cancerous tissues strongly suggest an important role in the pathobiology of the disease^8^. Based on this literature, the aim of our study was to investigate the high levels of HA and understand the role of HA in the pathobiology of gastric cancer. Future treatment of cancer depends on our ability to target cell proliferation and angiogenesis in this disease. We think that HA may have an effective role in carcinogenesis and believe that our study will be useful to understand the pathobiology of gastric cancer as well as to develop better ways for diagnosis, follow-up, and treatment of the disease.

## METHODS

### Collection and storage of blood and tissue samples

Blood samples were collected in ethylenediaminetetraacetic acid (EDTA) tubes from 82 gastric cancer patients and 41 healthy volunteers and centrifuged at 1,500 rpm for 15 min. The plasma portion of the blood was stored at −80°C indefinitely until all samples were collected. Gastric cancer (n=27) and healthy (n=29) tissues obtained after surgery were each divided into three parts. The first piece was frozen directly and stored at −80°C until the day of the experiment to be used to measure protein levels (Western blotting). The second piece was fixed by placing it directly in a 10% formaldehyde solution and placed on paraffin blocks to take tissue sections as detailed below. The third part was taken directly into 1 mL Trizol and stored at −80°C for messenger RNA (mRNA) extraction. The study was approved by the Erciyes University Ethics Committee approval (#2013/580) in accordance with the Declaration of Helsinki.

### Measurement of plasma hyaluronan levels by enzyme-linked immunosorbent assay

Plasma HA measurements were performed with the aid of commercially purchased enzyme-linked ımmunosorbent assay (ELISA) kits (catalog no. DY3614; R&D Systems). The experiment was carried out according to the manufacturer's protocol. All products from the kit were kept at room temperature for 30 min before use. Plasma samples were read in a ThermoMultiskan™ GO Microplate Spectrophotometer microplate reader at 450 nm wavelength, and the results were expressed as ng/mL.

### Protein level measurement (western blotting)

The obtained tissues were lysed in the lysis buffer (50 nM Tris, pH 7.4, 100 mM NaCl, 1 mM EDTA, 1% Nonidet P-40, and 10% glycerol together with 10 μg/mL pepstatin, 1 mM phenylmethylsulfonyl fluoride (PMSF), 1 mM dithiothreitol (DTT), 20 μg/mL aprotinin, 5 μg/mL leupeptin, and 1 μM sodium orthovanadate) using a homogenizer. Lysates were incubated with 1% SDS-PAGE buffer containing 10% 2-mercaptaethanol for 5 min at 95°C. The prepared proteins were run on 8% SDS-PAGE, and the proteins from the gel were transferred to the nitrocellulose membrane. The membrane was blocked with 5% skim milk powder for 1 h at room temperature (RT), followed by primary antibody incubation (1 h at RT) against the protein of interest. After incubation, the membrane was washed three times for 15 min in phosphate-buffered saline containing 0.02% Tween 20. After incubation, the washed membrane was incubated with 1/2,500 secondary antibody for 1 h at RT and washed three times for 15 min after incubation. Protein bands were detected by the BIORAD gel documentation system device by applying enhanced chemiluminescence for 1 min to the washed membrane.

### Immunofluorescence staining

Tissues taken for histological examination were immediately fixed with a 10% formaldehyde solution and then dehydrated by passing through a graded series of ethyl alcohol. Tissues cleared with xylol were embedded in paraffin. Sections (5 μm) were cut from paraffin blocks and placed on poly-lysine slides. After the sections were kept in an oven at 60°C for 2 h, the paraffin was removed by washing and passing through xylene as well as a decreasing graded alcohol series (100, 96, 70, and 50%). Tissues were then blocked for 30 min in Hank's buffer containing 2.0% bovine serum albumin (BSA), followed by overnight incubation at +4°C with a primary Ab added to Hank's buffer containing 2% BSA. Tissues were washed three times for 5 min with Hank's buffer (without BSA) and incubated for 45 min at RT, followed by secondary antibody incubation. After washing with BSA-free Hank's buffer three times as above, a drop of DAPI (VectaShield; VectorLabs) was dripped onto the sections, covered with a coverslip, and stored at −80°C until examined under a microscope.

### Real-time quantitative reverse transcription polymerase chain reaction for messenger RNA expression

TSG-6 mRNA expression was measured by real-time quantitative polymerase chain reaction (PCR) with cDNA obtained from both cancerous gastric tissues and healthy gastric tissues. RNA was isolated from tissues by the Trizol method and DNase I was added to the obtained RNA to avoid any DNA contamination.

cDNA was obtained from total RNA (a maximum of 1 μg RNA was used) by RT-PCR using the SuperScript First-Strand Synthesis System according to the manufacturer's recommendation. The following protocol was then applied: 1 μg RNA+1 μL Oligo(dT)+1 μL 10 mM dNTP mixture was made up to 10 μL and incubated at 65°C for 5 min for denaturation, and incubated on ice to bind primers. Then, add 10 μL of cDNA synthesis mix (10× RT buffer 2 μL, 25 mM MgCl_2_ 4 μL, 0.1 M DTT 2 μL, RNaseOUT (40 U/μL), 1 μL SuperScript III RT (200 U/μL) 1 μL) was added and incubated at 500°C for 50 min. After the reaction was terminated by incubation for 5 min at 85°C, the 20 μL cDNA reaction mixture obtained was diluted 10-fold and used in real-time PCR.

mRNA expressions of the TSG-6 enzyme were measured by real-time quantitative PCR from gastric tissues obtained from both gastric cancer and healthy individuals. RNA was isolated from tissues by the Trizol method, and DNase I was added to the obtained RNA to avoid any DNA contamination. cDNA was obtained from total RNA (a maximum of 1 μg RNA was used) by RT-PCR using the SuperScript First-Strand Synthesis System according to the manufacturer's recommendation. For all real-time PCR, SYBR green technology was used with the following conduction. The PCR reactions were placed on 96-well plates and amplified using the two-step fast PCR protocol enabled by the Roche instrument. The amplification plots were analyzed using the instrument software, and then the gene expression quantification was done by the method ΔΔCt calculation^9^.

### Statistics

All statistical analyses were performed with the JUMP (JMP version: 5.0.1.2 program). Continuous variables were compared with an independent two-tailed t-test. A p<0.05 was considered statistically significant.

## RESULTS

### Plasma hyaluronan levels

**Figure 1:**
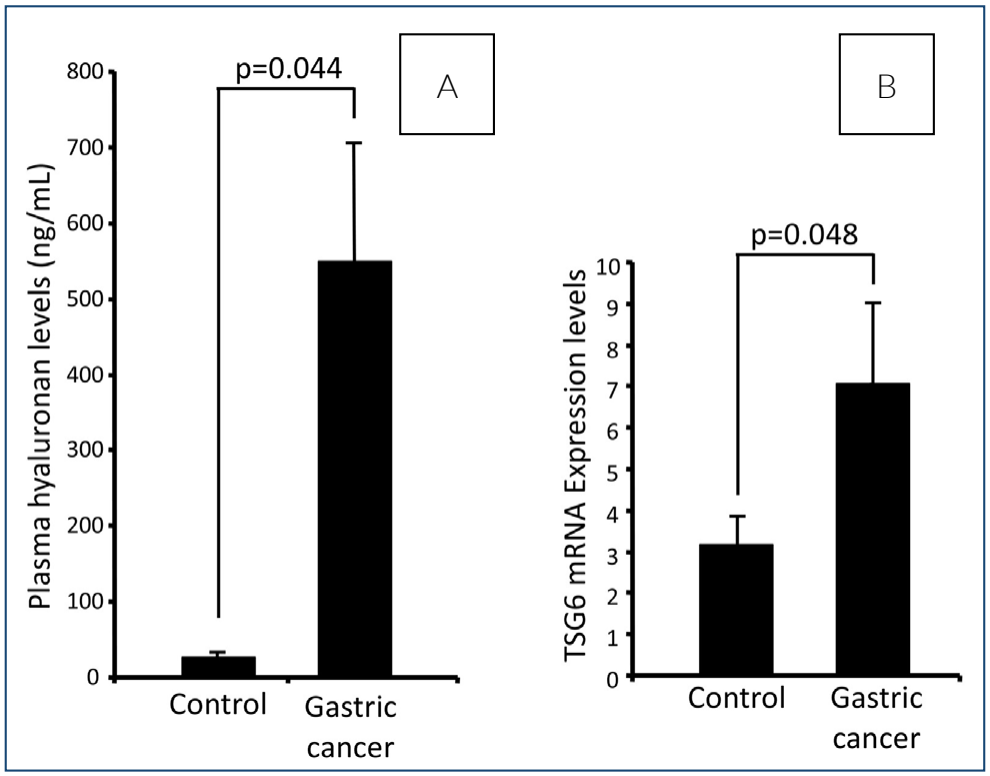
Plasma hyaluronan levels (A) and tumor necrosis factor-inducible gene 6 messenger RNA expression (B) with room temperature-polymerase chain reaction in patients with gastric cancers and controls.

According to the results, there was a statistically higher amount of HA in the blood of cancer patients compared to controls. Plasma HA levels mg/dL (mean±SD); gastric cancer (n=82): 549.80±155.68, healthy control (n=41): 27.21±4.95 (p<0.044) (Figure 1).

### Tissue hyaluronan staining

**Figure 2:**
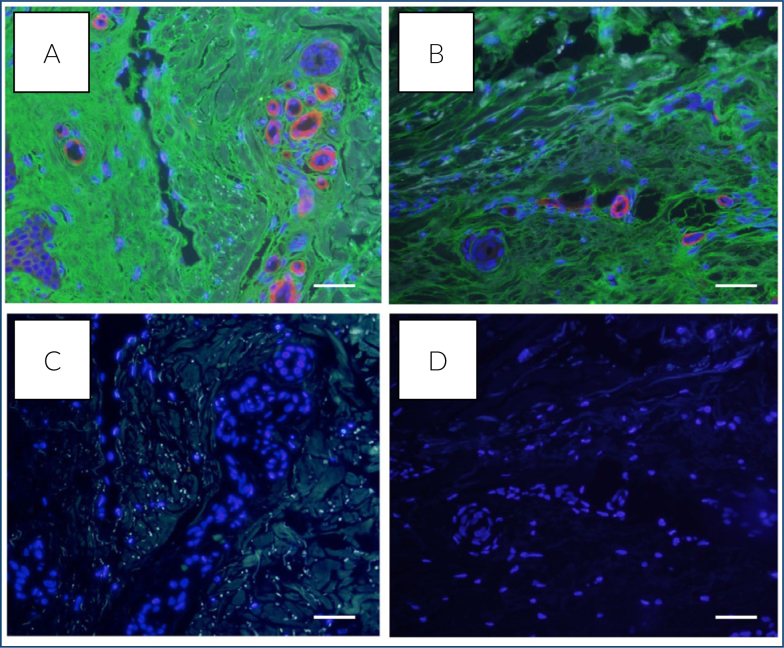
Intense hyaluronan staining was observed in gastric cancerous tissues (A) compared to non-cancerous tissues (B) with hyaluronan-binding protein immunofluorescence staining (green). DAPI stained nuclei (blue). (C) and (D) were recorded as negative controls using only secondary antibody. Scale bar=100 μm, 20× magnification.

HA-binding protein was stained by immunofluorescence staining. When both gastric cancerous and non-cancerous tissues were stained, HA deposition was observed to accumulate much more in cancerous tissues than in non-cancerous tissues (Figure 2).

### Messenger RNA expression levels of tumor necrosis factor-inducible gene 6

**Figure 3:**
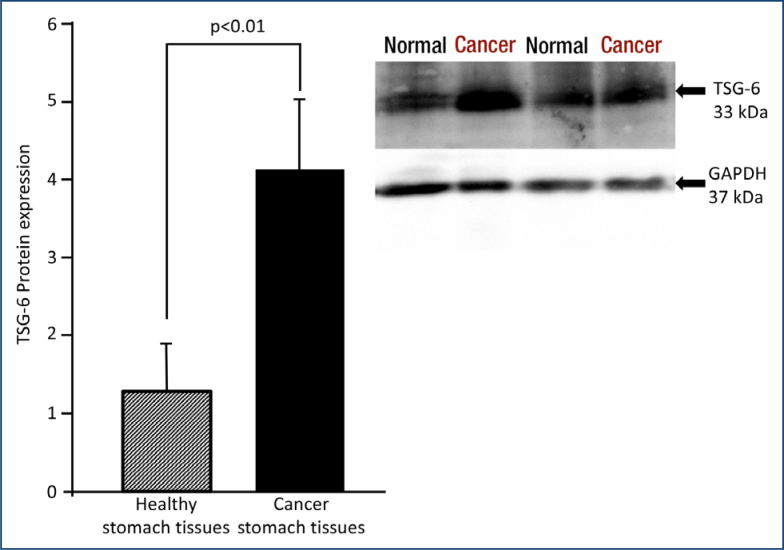
Tumor necrosis factor-inducible gene 6 protein expression is higher in gastric cancer as compared to normal tissues.

Accordingly, TSG-6 enzyme mRNA expression was measured in gastric cancer and healthy control tissues. TSG-6 mRNA expression was found to be statistically and significantly increased in gastric cancer tissues compared to healthy controls [TSG-6 mRNA expression; gastric cancer (n=27): 7.09±1.94, healthy control (n=29): 3.20±0.67 (p=0.048)] (Figure 3).

### Tumor necrosis factor-inducible gene 6 protein levels

TSG-6 protein expression was found to be higher in cancerous gastric tissues compared to control gastric tissues according to Western blot analysis (Figure 3). Thus, TSG-6 protein expression (mean±SD) was found to be 4.12±0.9 in gastric cancer (n=30) and 1.28±0.6 in healthy controls (n=30) (p<0.01).

## DISCUSSION

Many studies are carried out to reveal the mechanism of serious fatal cancer diseases. Especially in recent years, studies showing that the ECM profile has a role in the progression of cancer are included in the literature^10^. It has been reported that ECM has important roles in the proliferation and migration of cells^11^. There are also studies showing that some cancer types, including gastric cancer, are associated with ECM abnormalities^10,12^. In this respect, our study is important because identifying ECM abnormalities in gastric cancer will help us understand the process of the disease. It is known that matrix components are also related to the regulation of physiological and pathological processes in addition to their participation in the organ structure^13^. Apart from this, the presence of studies showing that the degraded products of the HA molecule also stimulate angiogenesis indicates that it is highly likely to play a role in gastric cancer as well^4^. In our study, the detection of a much higher amount of HA in the peripheral blood samples of gastric cancer patients compared to the control group confirms the results in the literature. In this respect, peripheral blood levels of HA seem to have the potential to be a tumor marker^14^. At the same time, the detection of HA accumulation in the immunofluorescence staining results of the tissues, especially in the cancerous tissues, suggests that HA has a potential role in the formation of the disease.

TSG-6 enzymes provide modification of hyaluronan by adding heavy chains onto HA. As a result of this event, there are studies showing that modified hyaluronan also causes it to take on different tasks^15^. Unlike other studies, in our study, both mRNA and protein expression of the TSG-6 enzyme were examined. Our demonstration of significantly higher TSG-6 mRNA and protein expression in cancerous gastric tissues compared to control gastric tissues suggests that HA modification is most likely present in gastric cancer.

The high gene expression of TSG-6 in tissues with gastric cancer also indicates that one or more cell populations are actively producing this enzyme. The abbreviation TSG-6 stands for "tumor necrosis factor-induced gene-6" because it was originally identified as a novel gene induced in human fibroblasts treated with TNF-alpha^16^. Therefore, smooth muscle cells may be the source of TSG-6 in gastric tissue. Since our study is the first to show the TSG-6 enzyme in gastric cancer tissues, more studies are needed to determine the etiology of TSG-6 in gastric cancer. We present evidence that TSG-6 is found at higher levels in gastric cancer tissue than in normal tissue in terms of both mRNA and protein levels. This also suggests that high levels of TSG-6 and HA in gastric cancer may drive cell proliferation and vascular remodeling.

When the effects of ECM components on cancer formation are revealed, we hope that a mechanism for cancer prevention or halting progression will be uncovered. Considering that the HA modification affects cell proliferation, blocking this modification in patients with gastric cancer may inhibit cell proliferation and stop cancer progression. This mechanism will become clearer with further studies in light of the data in our study.

## Data Availability

The data that support the findings of this study are available from the corresponding author upon reasonable request.
